# Crusted, Chronic, and Confusing

**DOI:** 10.1093/cid/ciaf583

**Published:** 2026-05-20

**Authors:** Luisa Hurtado-Rossi, Jonny A García-Luna, Hendrik Sy, Alvaro J Martinez-Valencia

**Affiliations:** Centro Internacional de Entrenamiento e Investigaciones Médicas (CIDEIM), Cali, Colombia; Centro Internacional de Entrenamiento e Investigaciones Médicas (CIDEIM), Cali, Colombia; Universidad Icesi, Cali, Colombia; Department of Infectious Diseases, Albert Einstein College of Medicine, Bronx, New York, USA; Centro Internacional de Entrenamiento e Investigaciones Médicas (CIDEIM), Cali, Colombia; Universidad Icesi, Cali, Colombia; Fundación Valle del Lili, Departamento de Medicina Interna, Servicio de Infectología, Cali, Colombia

## Abstract

Chronic ulcerative lesions initially treated as bacterial cellulitis were ultimately diagnosed as cutaneous leishmaniasis. This case highlights diagnostic pitfalls in persistent ulcers, the importance of considering endemic exposures, and the role of species identification in guiding therapy.

## QUESTION

**Figure 1.**
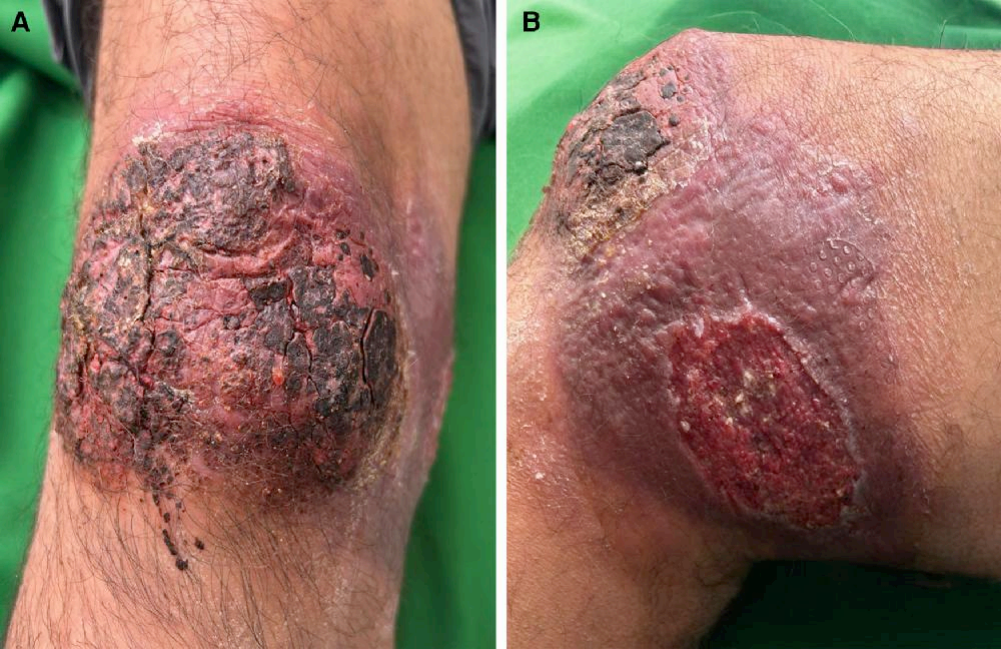
*A and B*, Ulcerative skin lesions at presentation. *A*, Thick crusted plaque with irregular raised borders involving the anterior surface of the right knee. *B*, Satellite ulcer with granulation tissue and surrounding erythema on the medial aspect of the same knee. Both lesions had persisted despite multiple courses of systemic antibiotics for presumed bacterial cellulitis.

A 64-year-old man from Cali, Valle del Cauca, Colombia, presented with a 3-month history of progressive ulcerative lesions on his right knee. He had been hospitalized twice and treated empirically with intravenous antibiotics for presumed bacterial cellulitis. Wound swabs and debrided tissue cultures grew *Klebsiella pneumoniae* resistant to ciprofloxacin, leading to prolonged antibiotic therapy, including ertapenem, cefepime, and piperacillin/tazobactam. Despite transient improvement in inflammation, ulceration persisted. He denied fever but reported local warmth, discomfort when walking, and palpable nodules behind the knee. Examination revealed a large ulcerated plaque with an elevated border and thick overlying crust on the anterior right knee. The lesion was tender to palpation but did not limit joint mobility. A second ulcer with a granulating base and erythematous margins was noted on the medial aspect of the right knee (Figures 1*A* and 1*B*). Three enlarged, firm, mobile lymph nodes were palpated in the popliteal fossa. The patient recalled noticing two papular lesions on his right knee after insect bites during a 2-day stay at a family farm in Cisneros, Buenaventura (Valle del Cauca, Colombia). He reported feeling insect bites during the early morning hours, and the appearance of erythematous papules the same day. About two weeks later, the papules developed crusts, which evolved into ulcerated lesions. Laboratory testing included negative PCR for *Mycobacterium tuberculosis* complex and repeated negative liquid cultures. A Giemsa-stained smear of lesion exudate revealed intracellular microorganisms, and tissue culture was performed for species identification. What is your diagnosis?

Diagnosis: Cutaneous leishmaniasis due to *Leishmania (Viannia) braziliensis*

**Figure 2.**
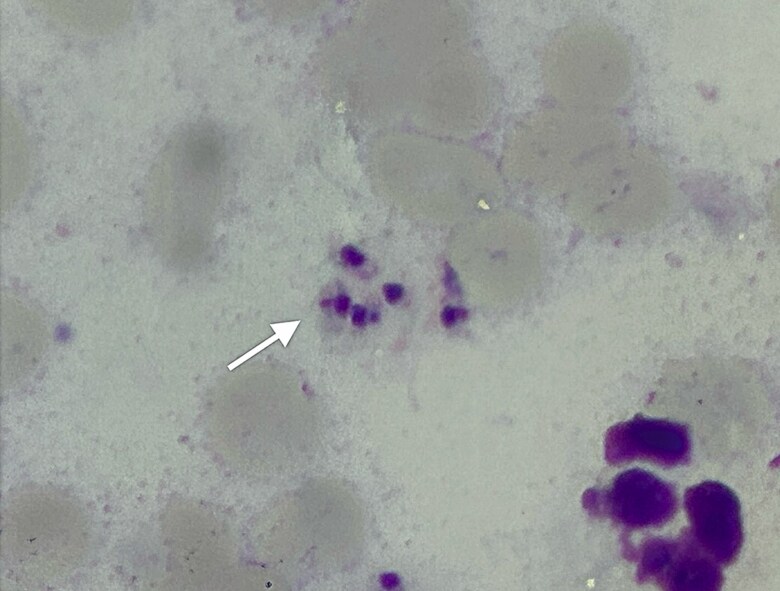
Intracellular Leishmania amastigotes in tissue smear. Giemsa-stained smear (×100 magnification, oil immersion) of exudate from the ulcer shows multiple intracellular amastigotes (arrow), consistent with cutaneous leishmaniasis.

This case describes a 64-year-old man who developed chronic ulcers on the right knee after visiting an endemic region in Colombia. Initial misdiagnosis as bacterial cellulitis led to treatment with multiple antibiotics following isolation of *K. pneumoniae* from wound cultures. Despite this, the lesions worsened. Giemsa-stained smear confirmed CL by demonstrating amastigotes (Figure 2). Species identification was performed culturing lesion tissue, followed by isoenzyme analysis, which identified *Leishmania (Viannia) braziliensis*.

Cutaneous leishmaniasis (CL) is a parasitic skin disease caused by protozoa of the genus *Leishmania*, transmitted through the bite of infected phlebotomine sand flies. According to WHO, CL accounts for 95% of cases in the Americas, the Mediterranean basin, the Middle East and Central Asia [1]. Some species of the subgenus *Viannia*, including *Leishmania braziliensis*, are associated with an aggressive clinical course and risk of mucosal dissemination, with an incubation period usually ranging from 2 weeks to 2 months. In this patient, papules appeared shortly after insect bites, with ulceration by two weeks, consistent with the short end of this spectrum and the rapid evolution characteristic of *L. braziliensis* infections [2]. Travelers, even during short-term stays in endemic areas, may be at risk when protective measures—repellents, protective clothing, or bed nets—are inconsistently used.

Chronic nonhealing ulcers are diagnostically challenging, as bacterial colonization may obscure the cause and lead to misdiagnosis, delaying treatment [3]. Assessment of epidemiologic exposures—such as insect bites or travel to rural areas—is essential. In this patient, chronicity, absence of systemic symptoms, antibiotic response, and epidemiologic context prompted evaluation for alternative infectious etiologies. Leishmaniasis in Colombia has a heterogeneous distribution: transmission occurs in rural areas, but not all regions are endemic. The Pacific region (Valle del Cauca, Cauca, and Nariño) presents a high risk due to abundant sand flies sustained by favorable ecological conditions [4]. Spatial models show clusters in rural municipalities, and major cities such as Bogotá and Cali are considered nonendemic.

The diagnosis was initially missed because the presentation suggested bacterial cellulitis rather than CL. Lesions caused by *L. braziliensis* evolved rapidly into large ulcers with marked erythema; the frontal lesion developed a prominent crust that masked typical features of localized CL. Isolation of *K. pneumoniae* from wound cultures reinforced this impression and led to prolonged antibiotic use. The patient was initially seen in Cali, a nonendemic city, and the epidemiologic clue of recent rural travel was not fully captured, further delaying recognition. Diagnosis of CL was established three months after symptom onset.

**Figure 3.**
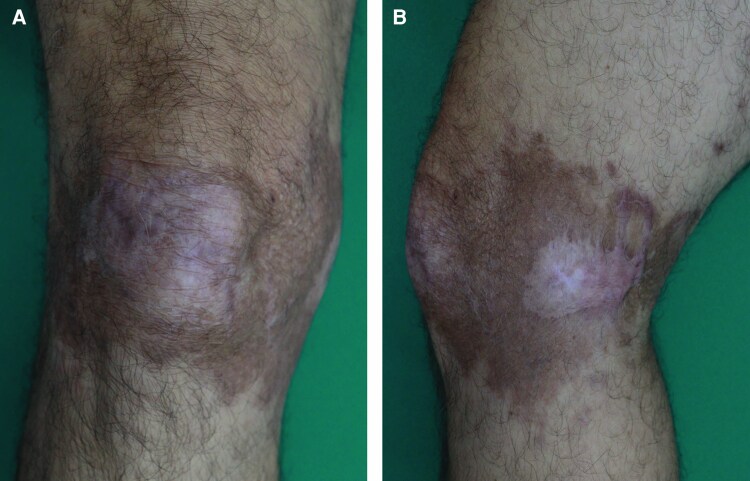
Clinical resolution following systemic antileishmanial therapy. *A*, Healed scar of the anterior knee plaque and (*B*) re-epithelialization of the medial ulcer after treatment with liposomal amphotericin B. Images obtained at 26 wks post-treatment initiation show complete lesion resolution and residual postinflammatory hyperpigmentation.

Given the patient's age and lack of mucosal involvement, miltefosine (50 mg TID for 28 days) was initiated while awaiting species results. By week 3, the lesions showed no improvement and satellite lesions appeared, suggesting treatment failure. Given the patient's age, comorbidities, and failure of initial therapy, intravenous liposomal amphotericin B (3 mg/kg on alternate days for 10 doses) was chosen as an alternative to antimonials. The patient was admitted for monitoring, including preinfusion laboratory testing, blood pressure surveillance, and thromboprophylaxis. Progressive healing was observed, with re-epithelialization by week 13 and complete resolution by week 26 (Figure 3).

Diagnosis of CL relies on clinical suspicion, parasitological methods (smear, histopathology, and culture) or molecular tools. Species identification is crucial in *L. braziliensis* infections due to risk of mucosal dissemination and variable treatment response [5]. PCR is more sensitive than microscopy, but cost, infrastructure, and need for trained personnel make it impractical in endemic regions; therefore, microscopy remains the most common diagnostic method [6]. Early species typing is essential for guiding therapy and preventing complications.

Miltefosine is the only oral antileishmanial agent approved in the Americas, but its efficacy against *L. braziliensis* is comparatively lower. Resistance involves reduced uptake due to mutations or inactivation of the LdMT-LdRos3 transporter, as demonstrated in resistant strains [7]. Clinical studies have documented treatment failure rates of ∼25% for *L. braziliensis* CL treated with miltefosine [8]. Liposomal amphotericin B has demonstrated efficacy in case series of travelers, including *L. braziliensis* infections, with cure rates up to 88%, and tolerability, supporting its role in severe or refractory cases [9].

This case highlights the importance of epidemiological history (travel to endemic areas or insect bites) and careful diagnostic evaluation. Misdiagnosis as bacterial cellulitis is common and can delay definitive treatment. IDSA and ASTMH recommend systemic therapy for *L. braziliensis* infections, even when lesions are localized, reducing the risk of mucosal involvement [10]. Clinicians must remain alert to treatment failure and consider alternative regimens when response to miltefosine is suboptimal.
